# Wound defects in the elderly: our experience

**DOI:** 10.1186/1471-2318-11-S1-A15

**Published:** 2011-08-24

**Authors:** A Ferrarese, V Martino, M Nano

**Affiliations:** 1 Department of Clinical and Biologic Science, School of Medicine and Surgery “S. Luigi Gonzaga” - AOU San Luigi Gonzaga – Orbassano , Turin - SCDU General Surgery University of Turin, Italy

## Background

We report our experience on clinical outcomes of elderly patients who have undergone laparoscopic repair for incisional and primary inguinal hernias.

## Patients and methods

To assess the safety and efficacy of laparoscopic [1-5] primary inguinal and incisional repair we reviewed the records of our patients of over 70 years old, who underwent such a procedure from June 2007 to September 2010: hernia defect size, recurrence, operative time, and procedure-related complications [6] were evaluated and a laparoscopic approach was attempted in all patients who required a mesh repair.

We scheduled 42 patients (32 M - 10 F, with 53 wound defects totally) for laparoscopic incisional [7] and primary inguinal hernia repair and we performed 17 surgical repair for incisional hernia and 36 for primary hernia. Of those, 13 were done for incisional hernias with a single defect (24.5% recurrence hernias), 4 with multiple defects (7.54% recurrence hernias), 12 were performed for unilateral inguinal hernias (22.56 % recurrence hernias), 16 for bilateral inguinal hernias (30.08 % recurrence hernias), 4 for umbilical hernias (1 recurrence hernias), 2 for epigastric and linea alba’s hernias, and 2 for rectum diastasis.

The majority of the patients were normal weight with a mean BMI of 25 kg/m2 (45%), 38% 25 > BMI > 30 (overweight), 17% BMI > 30 (obesity).

There was no conversion to an open procedure. The mean operative time was 128 minutes (range: 50 – 325).

In all the patients only mesh was used (37.5% polypropylene not reabsorbable, 42.5% tridimensional polyester-collagen composite mesh, 20% lightweight multifilament mesh partly reabsorbable) [8]. The meshes were fixed in 82.5 % with absorbable fixation device, in 5% with a non-absorbable device and in 12.5 % with fibrin glue [9].

In contrast to other authors [10-13], major complications were 14.24% (6/42: 2 chronic inguinal pain, 4 recurrences). Minor complications were 5/42 (11.90%) and included only asymptomatic seromas that were aspirated. The mean hospital stay was 4.7 days (range: 1-18 days).

## Conclusions

Laparoscopic repair of primary inguinal and incisional ventral hernias with transabdominal placement of composite mesh in the elderly achieves excellent results with low morbidity in comparison with open surgical approaches^(2,4,5)^. In our experience, adequate fixation of the mesh, extension to cover the entire previous incision and standardization of the placement interval of the sutures are crucial to the success of the repair.
